# Vertebrates can be more important pollinators than invertebrates on islands: the case of *Malva (=Lavatera) arborea* L.

**DOI:** 10.1093/aobpla/plae010

**Published:** 2024-03-01

**Authors:** Cristina Robles, Víctor Romero-Egea, Anna Traveset, Rocío Ruiz de Ybáñez, Sandra Hervías-Parejo

**Affiliations:** Department of Animal Health, Campus de Espinardo, University of Murcia, 30100 Murcia, Spain; Department of Animal Health, Campus de Espinardo, University of Murcia, 30100 Murcia, Spain; Mediterranean Institute for Advanced Studies (IMEDEA, UIB-CSIC), Global Change Research group, Miquel Marquès 21, 07190 Esporles, Spain; Department of Animal Health, Campus de Espinardo, University of Murcia, 30100 Murcia, Spain; Mediterranean Institute for Advanced Studies (IMEDEA, UIB-CSIC), Global Change Research group, Miquel Marquès 21, 07190 Esporles, Spain; Centre for Functional Ecology (CFE-UC), Community Ecology Lab, Department of Life Sciences, University of Coimbra, Calçada Martim de Freitas, 3004-531 Coimbra, Portugal

**Keywords:** Autogamy, birds, floral visitation, fruit set, insects, islands, lizards, pollinator exclusion experiments, reproductive system, seedling experiments, seed set

## Abstract

*Premise of the study:* On islands, flowering plants tend to be more generalist in their pollination needs, as insects (the main pollinators of flowering plants) are underrepresented in these ecosystems compared to the mainland. In addition, some vertebrate species that are typically insectivorous or granivorous on the mainland are forced to broaden their diet and consume other resources such as nectar or pollen on the islands. The shrub *Malva arborea*, with its large and colourful flowers, attracts different groups of potential pollinators. This study aimed to compare the effectiveness of vertebrates versus insects in an insular population of *M. arborea* and to investigate its reproductive system. *Methods:* For three groups of taxa (insects, birds and lizards), we assessed the two components of pollination effectiveness: (i) the quantitative component (i.e. number of visits and number of flowers contacted) through direct observations of flowers; and (ii) the qualitative component (fruit and seed set, number and size of seeds and proportion of seedling emergence) through pollinator exclusion experiments. *Key results:* Vertebrates (birds and lizards) were quantitatively the most effective pollinators, followed by insects. However, when all three groups visited the flowers, fruit and seed set were higher than when any of them were excluded. We also found that *M. arborea* has hermaphrodite flowers and is able to reproduce by autogamy, although less efficiently than when pollinated by animals. *Conclusions:* Both vertebrates and insects play an important role in the reproduction of *M. arborea*. Although the plant does not need pollinators to produce seeds, its reproductive success increases when all pollinators are allowed to visit the flowers. Besides providing new information on *M. arborea,* these findings may help to better understand the role of different pollinator groups in the reproduction of other plant species, especially on islands where the co-occurrence of vertebrate and invertebrate pollination in the same plant species is usual.

## Teaser

On islands, flowering plants may be more adaptable in their pollination requirements due to the scarcity of insects, which are the primary pollinators. In addition, vertebrates that typically feed on insects or seeds on the mainland also consume nectar or pollen on islands. This study shows that both vertebrates and insects are attracted to the large, showy flowers of *Malva arborea* , which can self-pollinate, although less efficiently than through animal pollination. The combined pollination system may explain its wide distribution on Mediterranean islands.

## Introduction

One of the most relevant plant–animal interactions for ecosystem functioning is that between angiosperms (flowering plants) and their pollinators. Without pollinators, approximately a third of angiosperm species would not be able to reproduce (Rodger *et al.* 2021). In addition, without these plants, pollinator populations would be diminished due to the lack of floral resources (pollen, nectar and floral water). There would also be knock-on effects for other species as a result of the loss of pollinators (Kearns *et al.* 1998; Ollerton *et al.* 2011; Ortega-Olivencia *et al.* 2012; Benton *et al.* 2022).

On islands, specialized pollinators are typically underrepresented, particularly insects, and plants tend to be more generalized in their pollination requirements compared to mainland relatives (Barrett 1996, Traveset *et al.* 2016). Some vertebrate species, for example, birds and lizards, which are usually insectivorous or granivorous on the mainland, are forced to also consume nectar and pollen on islands, where resource availability is usually limited (Grant and Grant 1981; Olesen and Valido 2003; Valido *et al.* 2004; Traveset and Nogales 2015). By feeding on floral resources, these opportunistic vertebrates can act as effective pollinators increasing plant reproductive success (e.g. Rodríguez-Rodríguez *et al.* 2013; Hervías-Parejo and Traveset 2018).

Not all animals that visit flowers are pollinators, nor do all pollinators provide the same pollination effectiveness to the plant (Ne’eman *et al.* 2010; Ballantyne *et al.* 2015; Gorenflo *et al.* 2017; Hervías-Parejo and Traveset 2018). Thus, the quantitative component of pollination effectiveness (hereafter QNC, i.e. number of visits and number of flowers contacted, may or may not be correlated with the qualitative component of pollination effectiveness (hereafter QLC, i.e. fruit and seed set and size) (Mayfield *et al.* 2001; Rodríguez-Rodríguez and Valido 2008; Gorenflo *et al.* 2017; Hervías-Parejo and Traveset 2018)). Therefore, the two components (QNC and QLC) should be simultaneously studied to adequately compare the effectiveness among different pollinator groups (Schupp *et al.* 2017).

Previous studies have corroborated the importance of opportunistic birds in plant reproduction on islands, demonstrating the pollination effectiveness of native granivorous/frugivorous and insectivorous birds, despite the low frequency of flower visits compared to insects (Traveset *et al.* 2015; Hervías-Parejo and Traveset 2018; Olesen *et al.* 2018). However, very few studies have compared the pollinator effectiveness among insects, birds and lizards (but see Ortega-Olivencia *et al.* 2012; Rodríguez-Rodríguez *et al.* 2013; Jaca *et al.* 2018). The present study contributes to fill this gap of information.

*Malva (=Lavatera) arborea* is a widespread species of the Holarctic floral kingdom, characterized by being the largest Malvaceae plant on the islands (up to 3 m in height). Despite its wide distribution, there is a lack of knowledge on its reproductive system and the identity of its pollinators. Previous observations had suggested us that this plant—with large, conspicuous and easily accessible flowers—might be pollinated by different vertebrates (specifically, birds and lizards) as well as by insects and, thus, would be an ideal case study to compare their pollination effectiveness. Our previous observations also show that the plant produces two types of flowers in the same individual, one type with a more developed stigma than the stamens and another type with more developed stamens than the stigma, which suggested some kind of herkogamy or chasmogamy. Hence, the specific objectives of the study were: (i) to assess whether birds and lizards act as effective pollinators by comparing their QNC and QLC with those of insects; and (ii) to investigate the reproductive system of *M. arborea*, unknown so far. We hypothesized that the large and showy flowers of *M. arborea* would attract vertebrates that act as effective pollinators. Therefore, their exclusion would result in a decrease in the QLC components. The two quantitative subcomponents were used to plot the location of insects, birds and lizards on a quantitative component landscape (sensu Schupp *et al.* 2010, 2017).

## Material and Methods

### Study area

The study was conducted on the main island of Cabrera (Cabrera Gran, 39°08ʹ31″N 2°56ʹ45″E), the largest island of the Cabrera Archipelago Maritime-Terrestrial National Park (Balearic Islands). It is characterized by hosting the best-undisturbed island ecosystems of the Spanish Mediterranean, and by its various international recognitions, such as Special Protection Area for Birds (SPA), Site of Community Interest and Special Protection Area of Mediterranean Importance (CAIB 2022; MITECO 2022). The climate of Cabrera is of the semi-arid Mediterranean type, with dry and hot summers and mild winters with little rainfall. Average annual temperatures and precipitation are 17 °C and 434 mm, respectively (AEMET 2022; MITECO 2022).

### Study species

*Malva arborea* is found throughout the Western Mediterranean, reaching various points in Western Europe north to Ireland (Dalby 1968). Its habitats tend to be near the sea, such as rocky shores, beaches and cliffs where seabirds nest, but it can also be found in disturbed areas such as fields and roadsides. Its conservation status is of Least Concern according to the IUCN, due to its wide distribution and the abundance of its wild populations. The flowers of this woody base species have a conspicuous corolla consisting of five petals of pink or purple colour, up to 2 cm long, with dark veins (see http://www.floraiberica.es/floraiberica/texto/pdfs/03_060_12_Lavatera.pdf). Its androecium contains numerous stamens with filaments welded into a tube through which the style passes. The gynoecium consists of a cup-shaped ovary with numerous carpels in a single whorl, each with a seminal rudiment and the elongated stigmatic zone (WFO 2023). The populations of *M. arborea* studied are located at sea level, within the harbour of Cabrera Gran.

### Pollinator censuses

Direct observations of visits to *M. arborea* flowers by pollinators were made between approximately 8:45 a.m. and 11:45 p.m., encompassing the day and night interval during which both diurnal and nocturnal pollinators are active (total observation period = 22.5 h), from 5 April to 6 May, under favourable weather conditions, that is, on sunny and windless days. During each census that lasted 10 min, we recorded (i) all legitimate visits (i.e. the visitor entered the corolla and touched the reproductive organs), (ii) the visitor species, (iii) the number of flowers visited, (iv) the number of flowers observed during the census and (v) the number of flowers available in the plant. The observer (C.R.) was always at a distance of about 4–5 m away from the plant to decrease pollinator interference. Photographs of flower visitors were taken whenever possible. Once the census was completed, flowers were inspected at close range to photograph insects for later species identification. Insect identifications were made using a pollinator reference collection.

### Pollinator exclusion experiments

**Figure 1.**
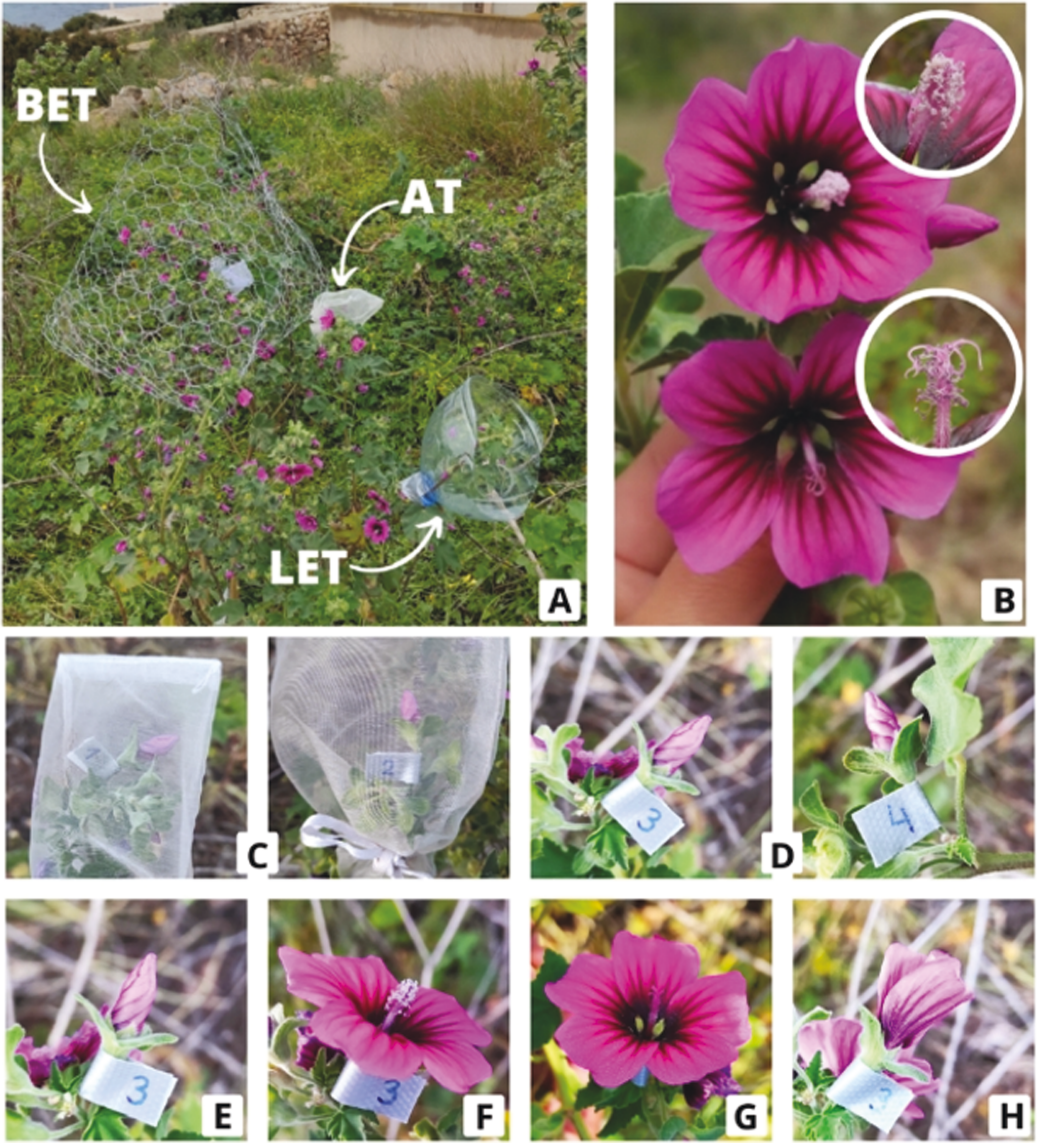
(A) Flowers of *M. arborea* with pollinator exclusion treatments: AT (autogamy treatment), LET (lizard exclusion treatment) and BET (bird exclusion treatment). (B) Morphologically female flower and morphologically male flower. Study of the reproductive system of *M. arborea:* (C) flowers with AT and (D) flowers with control treatment. Study of floral anthesis of *M. arborea*: (E) flower bud marked on 24 April at 8:40 p.m.; (F) flower opening on 25 April at 8:40 p.m; (G) changes observed in the flower on 26 April at 8:40 a.m.; (H) flower closed on 26 April at 20:40 p.m.

To assess the pollination effectiveness of the different flower visitors, pollinator exclusion experiments (Fig. 1) were conducted in April 2022 on 11 individual *M. arborea* plants of a similar size. For each individual, plant height and width were measured (as these variables might be relevant for pollinators when choosing the flowers to visit), and a similar number of flower buds were randomly assigned to four different treatments:

- Control treatment (CT), in which the flowers were left open to be visited by any pollinator.
- Autogamy treatment (AT), in which pollinators were completely excluded by placing a cloth bag around the flowers to test the plant ability to self-pollinate.
- Bird exclusion treatment (BET), in which a 25 mm pyramid-shaped wire mesh allowed only insects and lizards to visit the flowers.
- Lizard exclusion treatment (LET), in which a plastic jug surrounding the flowers (Fig. 1A) allowed only insects and birds to visit the flowers from above.

Treatments AT, BET and LET were checked daily from 5 April to 6 May 2022 to ensure that they were set correctly and excluded all pollinators, birds and lizards, respectively.

After the fruit ripening period, the fruits in each treatment were collected and counted to estimate fruit set (= number of fruits/number of buds). Whenever possible, five fruits per individual plant and treatment were randomly selected, and the number of seeds produced per fruit (seed set) was recorded. The maximum length of each seed was measured using a digital caliper, with an accuracy of 0.01 mm. Five seeds from each individual and treatment were also randomly selected for subsequent germination experiments in a greenhouse. The seeds were randomly arranged in space and sown individually in wells filled with organically fertilized peat moss mixture and constant humidity. The germination experiment took place from December 2022 to June 2023, until no seeds had germinated for a period of 30 days. Seedling emergence was recorded daily. The results are expressed as (1) seedling emergence percentage (i.e. percentage of total seeds that emerged) and (2) seedling emergence time (i.e. mean number of days to seedling emergence per treatment).

### Reproductive system of *Malva arborea*

To test whether the two flower types (Fig. 1B) of *M. arborea* produced fruits, 5 flowers of each type were marked as controls (CT) and 10 flower buds were excluded with the AT in 5 additional individuals (different from those on which the pollinator exclusions were performed). Since the flowers must be hermaphroditic for self-pollination to occur, these AT flowers were used to test whether the maturation of the two reproductive organs overlapped. These experiments were checked daily from 20–28 April until fruit set (Fig. 1C, D).

Finally, to assess whether anthesis (the time the flower remains open) of *M. arborea* varies between flower types and whether it can be affected by pollination exclusion, in the same five individuals we marked two flower buds as controls (CT) and excluded two others (AT) (Fig. 1E-H). The experiment lasted 4 days, from 24 to 27 April, and was checked every 12 h, at 8:40 a.m. and at 20:40 p.m.

### Data analysis

Differences in quantitative effectiveness (QNC) among pollinators were assessed using a generalized linear model (GLM) with each subcomponent (number of visits and number of flowers contacted) as a dependent variable in a separate model, and pollinator type (insect, bird or lizard) as a fixed factor. A GLM was performed using the ‘glm’ function (quasipoisson family) of the R package ‘stats’ (R Core Team 2023), followed by post hoc pairwise comparisons using the R package ‘emmeans’ (Lenth 2023).

Differences in qualitative effectiveness (QLC) among pollinators were assessed using generalized linear mixed models (GLMM) by including each subcomponent as a dependent variable in separate models: fruit set was fitted to a binomial distribution, seed set and seed size to a gamma distribution, seedling emergence percentage to a binomial distribution and seedling emergence time to a Poisson distribution. Pollinator exclusion treatment was included in the models as fixed explanatory variable, plant height and width as covariates, and individual plants and replicates as nested random effects to account for inherent differences between individuals and replicates. GLMMs were performed using the ‘glmer’ function of the R package’lme4’ (Bates *et al.* 2015), followed by Tukey’s post hoc tests using the ‘glht’ function of the package’multcomp’ (Hothorn *et al.* 2008). All statistical analyses were performed using the R program (v. 4.1.2) (R Core Team 2023).

## Results

**Figure 2.**
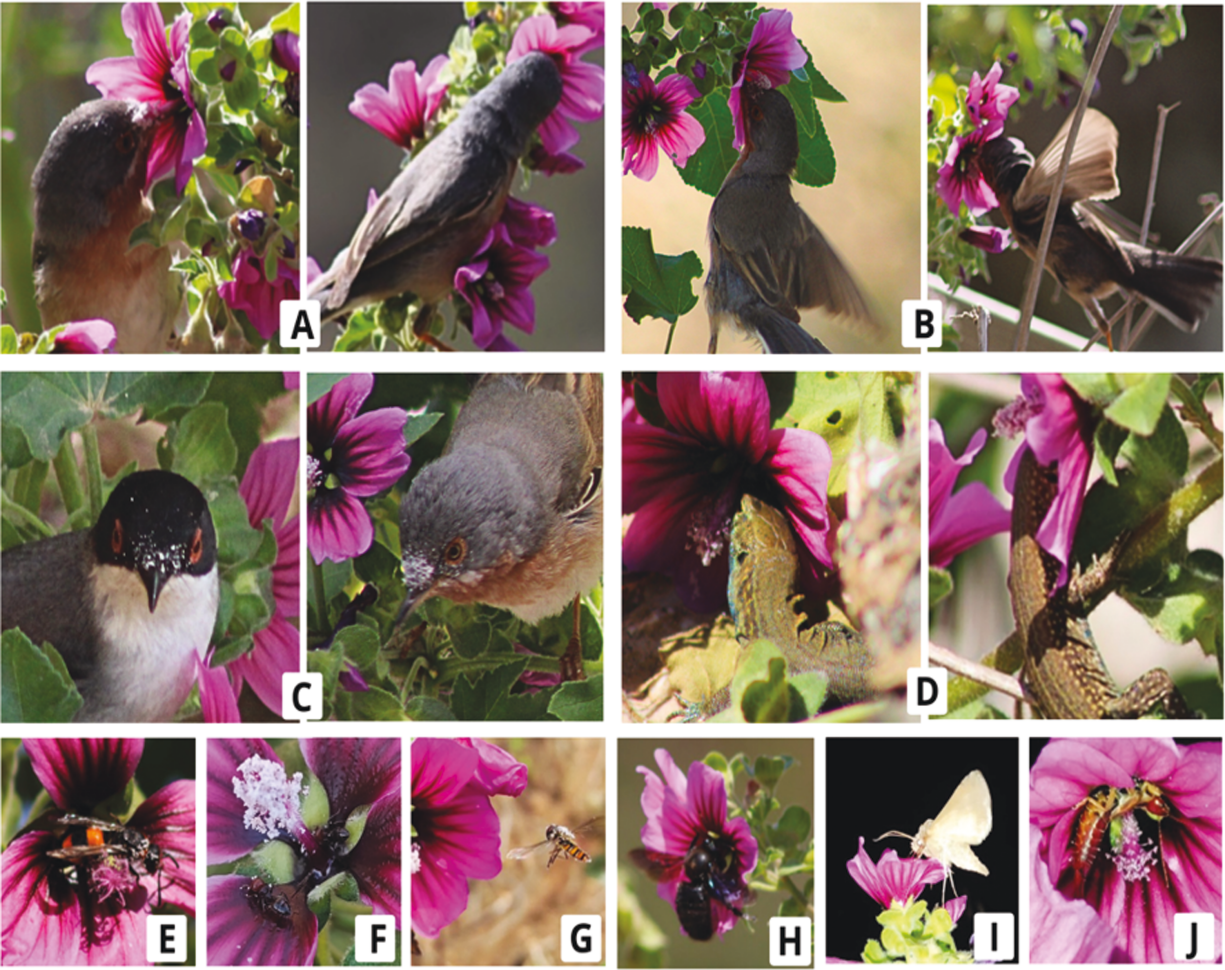
Flower visitors of *M. arborea* were observed during the censuses. (A) *S. cantillans*; (B) brief fluttering moments of *S. cantillans* during visits to flowers. (C) *Sylvia melanocephala* (left) and *S. cantillans* (right) with pollen (white spots) of *M. arborea*. Flowers of *M. arborea* visited by (D) *P. lilfordi*; (E) *Ancistrocerus ebusianus*; (F) *Crematogaster scutellaris*; (G) *Episyrphus balteatus*; (H) *Xylocopa violacea*; (I) *Mythimna* cf*. obsoleta* and (J) *Forficula auricularia.*

In total, we recorded 17 diurnal and 3 nocturnal insect species belonging to 5 orders: Hymenoptera (ants, wasps, bees and bumblebees), Diptera (flies and hoverflies), Thysanoptera, Lepidoptera (moths) and Dermaptera (earwigs) (Table 1); 3 passerines (*Sylvia cantillans*, *S. atricapilla* and *S. melanocephala*) and the Balearic lizard *Podarcis lilfordi*. All visits by insects and vertebrates were legitimate and are, therefore, considered potential pollinators. None of them were observed damaging the flower or robbing the nectar. One of the insect species observed is endemic to the Balearic Islands: the wasp *Ancistrocerus ebusianus*. Two ant species showed the highest visitation rate (*Monomorium salomonis* and *Tapinoma* cf. *nigerrimum*), followed by one species of thrips *Aeolothrips* sp. As for the bird floral visitors, all three species belong to the genus *Sylvia*, with *S. cantillans* being the most frequent visitor. Regarding lizard visits to flowers, they were mainly made by juveniles (50 %) and females (37.5 %) (Fig. 2).

**Table 1.** Mean values (± standard deviation) of the number of visits per 10 min and per individual plant and number of flowers contacted during a census, standardized by number of flowers observed, number of census per plant species and specific flower availability during the flowering period in 2022 on Cabrera Grand (Balearic Islands).

Species	Family	Functional group	*N* visits	*N* flowers contacted
**Insects**				
* Anthophora* cf*. subterranea*	Apidae	Hymenoptera	8.38 ± 6.38	4.83 ± 2.48
* Xylocopa violacea*			2.00 ± 1.73	2.00 ± 1.73
* Camponotus* sp.	Formicidae		6.00 ± NA	2.00 ± NA
* Crematogaster scutellaris*			3.00 ± NA	1.00 ± NA
* Monomorium salomonis*			68.00 ± NA	2.00 ± NA
* Plagiolepis* sp.			1.00 ± NA	1.00 ± NA
* Tapinoma* cf*. nigerrimum*			28.80 ± 25.66	2.40 ± 0.89
* Ancistrocerus ebusianus*	Vespidae		3.07 ± 2.30	2.36 ± 1.36
* Calliphora vicina*	Calliphoridae	Diptera	2.00 ± NA	2.00 ± NA
* Pollenia leclercqiana*			2.00 ± NA	2.00 ± NA
* Dasyphora albofasciata*	Muscidae		1.00 ± NA	1.00 ± NA
* Megaselia* sp.	Phoridae		1.00 ± NA	1.00 ± NA
* Episyrphus balteatus*	Syrphidae		5.50 ± 4.95	2.50 ± 0.71
* Eupeodes corollae*			3.13 ± 1.89	3.13 ± 1.89
* Trupanea stellata*	Tephritidae		1.00 ± NA	1.00 ± NA
* Aeolothrips* sp.	Aeolothripidae	Thysanoptera	13.80 ± 16.38	2.69 ± 1.38
* Thysanoptera* sp.	Thripidae		1.83 ± NA	1.00 ± NA
* Mythimna* cf*. obsoleta*	Noctuidae	Lepidoptera	4.00 ± 2.83	2.50 ± 0.71
* Noctua pronuba*			1.00 ± 0.00	1.00 ± 0.00
* Forficula auricularia*	Forficulidae	Dermaptera	10.60 ± 7.19	3.00 ± 1.00
**Birds**				
* Sylvia atricapilla*	Sylviidae	Passeriformes	4.12 ± 2.51	3.20 ± 1.79
* Sylvia cantillans*			8.31 ± 9.81	6.11 ± 5.28
* Sylvia melanocephala*			7.69 ± NA	5.00 ± NA
**Lizards**				
* Podarcis lilfordi*	Lacertidae	Squamata	20.46 ± 28.21	5.75 ± 4.99

### Quantitative importance of floral visitors

**Figure 3.**
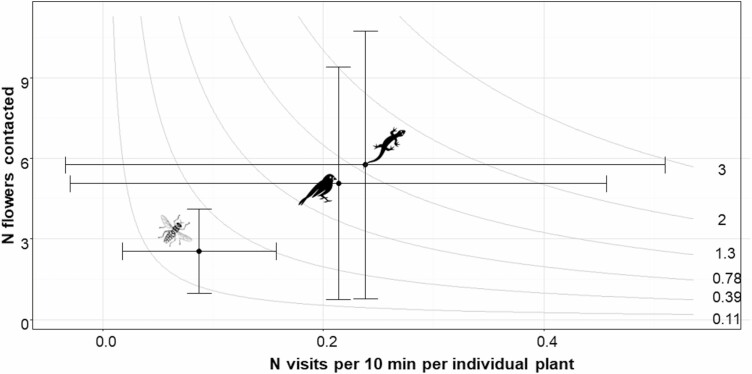
The distribution of insects, birds and lizards on the quantitative component pollination landscape for *M. arborea*. Isoclines represent all combinations of mean (± standard deviation) number of visits per 10 min and per plant and number of flowers contacted during a census, standardized by the number of flowers observed, number of census per individual plant and specific flower abundance during the flowering period in 2022 on Cabrera Grand (Balearic Islands).

Birds were observed visiting flowers 2.5 times more frequently than insects (*β* = 0.13 ± 0.04, *P* = 0.005). However, no significant differences were found in the number of visits between insects and lizards (*β* = −0.15 ± 0.08, *P* = 0.139) nor between birds and lizards (*β* = −0.02 ± 0.09, *P* = 0.958). Moreover, both birds (*β* = 0.15 ± 0.04, *P* = 0.002) and lizards (*β* = 0.19 ± 0.07, *P* = 0.037) contacted more flowers than insects, whereas no differences in the number of flowers contacted were found between birds and lizards (Fig. 3).

### Qualitative importance of floral visitors

**Figure 4.**
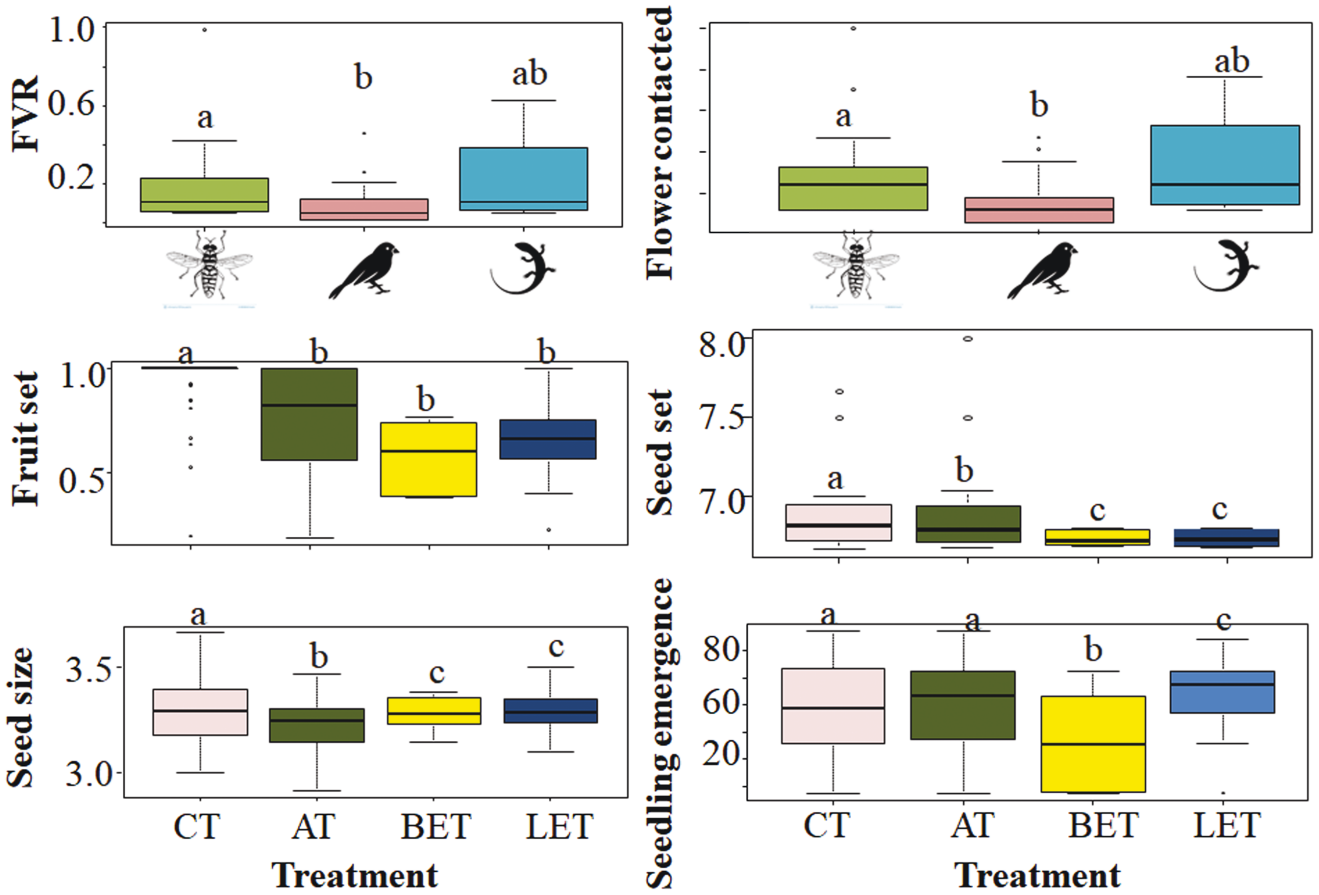
Mean values of fruit set, seed set, seed size and seedling emergence time for each treatment. Different letters indicate significant differences (*P* < 0.05).

Overall, control flowers showed higher fruit set and seed set and larger seeds than flowers from the three exclusion treatments (Fig. 4; Table 2). Autogamous flowers had higher seed set compared to the excluded flowers and gave smaller seeds than either excluded or control flowers. No significant differences were found between excluding birds and excluding lizards for any of the variables examined (Table 2). Furthermore, excluding birds resulted in a reduction of seedling emergence time by 0.7 compared to autogamous flowers, by 0.7 compared to control, and by 0.6 compared to excluding lizards, that is, the exclusion of birds produced seeds that emerged more slowly than the rest of treatments (Fig. 4). No differences between treatments were found in seedling emergence percentage.

**Table 2.** Results of the post hoc test to compare pollinator exclusion treatments two by two where each qualitative component of pollination effectiveness was used as a dependent variable.

Dependent variable	Treatment	*β*	Standard deviation	*Z*	*P*
Fruit set	CT-AT	0.7749	0.24	3.22	**0.0071**
	CT-BET	0.9294	0.23	3.95	**<0.001**
	CT-LET	0.8003	0.23	3.48	**0.0027**
	AT-BET	0.1545	0.22	0.69	0.9003
	AT-LET	0.0254	0.21	0.12	0.9994
	BET-LET	−0.1291	0.21	−0.62	0.9246
Seed set	CT-AT	0.0001	0.00	0.75	0.6102
	CT-BET	−0.0001	0.00	−2.82	**0.0236**
	CT-LET	−0.0001	0.00	−1.88	0.1792
	AT-BET	0.0002	0.00	3.39	**0.0041**
	AT-LET	0.0001	0.00	2.57	**0.0405**
	BET-LET	0.0001	0.00	1.03	0.6102
Seed size	CT-AT	0.0081	0.00	7.89	**<0.001**
	CT-BET	−0.0011	0.00	−0.89	1.0000
	CT-LET	−0.0005	0.00	0.44	**<0.001**
	AT-BET	−0.0069	0.00	−5.42	**<0.001**
	AT-LET	−0.0075	0.00	−6.09	**<0.001**
	BET-LET	0.0006	0.00	0.45	1.0000
Seedling emergence time	CT-AT	0.1952	0.04	4.82	<0.001
	CT-BET	0.4141	0.05	8.43	**<0.001**
	CT-LET	−0.1219	0.04	−3.19	**0.0014**
	AT-BET	0.2189	0.05	4.41	**<0.001**
	AT-LET	−0.3172	0.05	−6.98	**<0.001**
	BET-LET	−0.5361	0.05	−10.50	**<0.001**

### Reproductive system of *Malva arborea*

We found that flower anthesis lasts about 24 h, regardless flowers were bagged or not. When flowers open, the stamens always appear to be more developed than the stigmas and remain above them for about 12 h. At this time, the stigmas appear to be more developed and remain well above the stamens (Fig. 1B). At some point, there is simultaneous maturation of the male and female organs of the flower, as we confirmed that the species is capable of self-fertilization.

## Discussion

Our findings confirm that the pollination of *M. arborea* involves different taxa of pollinators, both insects and vertebrates that favour the reproductive success of the plant. This multiple pollination system is known in other plant species, such as *Scrophularia calliantha* and *Echium simplex* from the Canary Islands, which also have large and showy flowers and inflorescences, respectively, that are visited by insects, birds and lizards (Ortega-Olivencia *et al.* 2012; Jaca *et al.* 2018). Although *M. arborea* also seems to be able to reproduce by autogamy, its reproductive success is significantly higher when cross-pollination occurs.

### Effectiveness of *Malva arborea* pollinators

Among insects, Hymenoptera were the most frequent flower visitors, which is consistent with results obtained for other island Malvaceae species in the Americas (Sánchez Pérez 2013; Hazlehurst *et al.* 2023). To our knowledge, this is the first study of pollinators of *M. arborea* in its native range.

The high frequency of bird visits to flowers was from *S. cantillans*, endemic to the western Mediterranean and known to breed in Cabrera and Mallorca (Barcelo-Serra and Gordo 2022). In the vast majority of observations, a large amount of pollen could be seen adhering to the feathers on the front of the head (Fig. 2). Although most visits to flowers by this species tended to be made while perched, we were also able to confirm that they are capable of making short flights and hover to feed on flowers (Fig. 2). This behaviour had already been documented by Janeček *et al.* (2010), who argued to abandon the belief that only New World hummingbirds flit while Old World birds only feed while perched. Although detailed studies of vertebrate pollination in this species have not been conducted, our finding of effective pollination by generalist passerines is consistent with other island Malvaceae in the genus *Navaea* (Fernandez de Castro *et al.* 2017).

Most lizard observations were made on individuals of *M. arborea* a few centimetres high and/or, near walls or other rocky structures, which are used by lizards for shelter or to sun themselves in areas protected from the cooling effect of the wind (Pérez-Mellado *et al.* 2013). Pollination by reptiles (saurophilia) is a rare phenomenon that is believed to be associated with island ecosystems (and environments similar to those of islands) where they tend to expand their diet to include nectar and other floral resources (see review by Justicia-Correcher *et al.* 2023). The role of *P. lilfordi* species as a potential pollinator has in fact been recently documented at the plant community level in Cabrera Gran island (Romero-Egea *et al.* 2023).

Insects, birds and lizards all proved to be qualitatively effective pollinators for *M. arborea*. Their effectiveness was demonstrated by the greater number of fruits, seeds and seed size when insects, birds and lizards had access to the flowers compared to when one of them was excluded. This contrasts with the findings on *E. simplex* by Jaca *et al.* (2018), who found that vertebrates did not significantly contribute to increased plant fitness. The reduction in fruit set, seed set and seed length under vertebrate pollinator exclusion may indicate that *M. arborea* has evolved on this island together with birds and lizards as important pollinators, in addition to insects, as suggested by Hervías-Parejo and Traveset (2018) in their study with other island species. Among vertebrates, the role of birds may be more important than that of lizards, because the former move greater distances and, therefore, increasing genetic variability. Birds appear to play an important role in the reproduction of *M. arborea* by also reducing the time of seedling emergence, as concluded in the above study (Hervías-Parejo and Traveset 2018).

The high reproductive success of the ATs could be partly attributed to the fruit herbivory carried out by the larvae of a lepidopteran (*Heliothis armigera*), who feeds on the fruits once these are ripe, in addition to fruit consumption by rats (*Rattus rattus*). By bagging the flowers/fruits, we excluded these herbivores that were found to reduce fruit and seed set in the control treatments. A specific study on the rates of herbivory by these two pest species would be necessary to assess how important they are reducing the reproductive success of *M. arborea*.

### Reproductive system of *M. arborea*

Although morphologically the species appears to be dioecious (with female and male flowers), we found that it is monoecious or hermaphrodite with protandric flowers, that is, that function first as male and then as female flowers. This hermaphroditic state gives the plant a chance of reproducing either by cross- or self-fertilization (Barrett 2010). The disadvantage of self-fertilization is the poorer quality of the offspring and a significant reduction in their fitness compared to cross-fertilization. To reduce self-fertilization, strategies are often developed that separate the maturation of the plant sexes in space (herkogamy) or in time (dichogamy) (Webb and Lloyd 1986). Our findings show that the two types of flowers observed in the same individual respond only to a temporal asynchrony and, thus, that the strategy of *M. arborea* to reduce selfing is dichogamy.

### Concluding remarks

Our study provides the first evidence that vertebrates (three bird species of the genus *Sylvia* and the endemic lizard *P. lilfordi*) are effective pollinators of *M. arborea* and, quantitatively, they have more importance than insects. The high reproductive success of *M. arborea* may actually be explained by the combined pollination system, involving both insects and vertebrates. The hermaphroditic reproductive system and the ability to reproduce by autogamy probably also contribute to the high reproductive success of this shrub and explain its wide distribution in the Mediterranean islands. Overall, our results provide valuable information for a better understanding of the breeding strategies of island plant species and the role of different pollinator groups.

## Data Availability

The dataset is available at 10.6084/m9.figshare.25204958.
